# Editorial: Rational Design of Multi-Functional Nanomaterials

**DOI:** 10.3389/fchem.2019.00358

**Published:** 2019-05-24

**Authors:** Carlos Lodeiro, José Luis Capelo, Hugo M. Santos

**Affiliations:** ^1^BIOSCOPE Group, LAQV-REQUIMTE Research Unit, Chemistry Department, Faculty of Science and Technology, University NOVA of Lisbon, Caparica, Portugal; ^2^PROTEOMASS Scientific Society, Caparica, Portugal

**Keywords:** nanomaterials, nanoparticles, structural control, rational functionalization, detection, delivery, advanced multifunctional nanomaterials

At the beginning of 2018, and after closing the international symposium on Nanoparticles, Nanomaterials and Applications^1^ (3rd ISN2A) held in Costa de Caparica (Portugal), we encourage different research groups working at the nanoscale field to submit their latest scientific findings or revisions, to a new Research Topic devoted to “Rational Design of Multi-Functional Nanomaterials.” Two important keywords appear in the title: Rational-Design and Multi-functional Applications.

Concerning the first term, Rational Design, one of the most important subjects when a nanomaterial is designed, is to control the synthetic pathways to ensure the final desired product. A combination of dry and wet synthetic techniques, in conjunction with the adequate chemical and physical characterization methodologies, it is possible to prepare new nanomaterials successfully. This complex issue frequently is a result of multidisciplinary cooperation between chemists, physics, biologist, physicians, material engineers, among other disciplines. The second statement, Multifunctional applications, deals with the use of nanomaterials in fields some time collaboratives, such as drug delivery, cell and tissue imaging, as new antibiotic tools, in environmental detection and removing of contaminants, catalysis, and many industrial applications directly rely on properties such as water solubility, permeability, photostability, cell penetration, magnetic properties, related shape, and size control among others. Nowadays, functionalized nanomaterials play a crucial role in modern research areas because of their unique physical and chemical properties arise from their size and shape.

In the international year of the Periodic Table, we can highlight in this collection of papers the use of gold, silver, iron, platinum, molybdenum, titanium, and cobalt as metal precursors among the use of organic@inorganic polymers, and Silica as the chemical protagonist in this collection of papers.

Sixty-eight researchers from Portugal, Spain, Italy, Germany, Switzerland, Hungary, Russia, United States of America, Saudi Arabia, South Korea, Taiwan, and Indonesia, submitted 10 original and review articles covering all the aspects highlighted in the title. Related to the contributions in the field of biomedical sciences and applications, Caponetti et al. in Italy reported an elegant paper on Fluorescent Nanoparticles for bioimaging; The use of Gold Nanoparticles for Photothermal therapy was studied by Vines et al. in South Korea and the USA, and also Gold Based Organometallic nanocomposites for Antitumoral activity studies were reported by Dalmases et al. in a collaborative paper including researchers from Spain and Germany, and finally iron was ^1^ present as Magnetic Nanoparticles for drug delivery applications reviewed by Price et al. including researchers from the United States of America, Saudi Arabia, and Russia. The actually topic of antibiotic resistance and new antibiotic studies was covered using silver nanomaterials by Djafari et al. in Portugal with a manuscript about Silver Nanotriangles as antibacterial tools, and Nuti et al. with the contribution of researchers in Portugal, Spain, and Italy with the synthesis of Mesoporous Silver nanoparticles for antibiotic delivery.

Catalysis and industrial applications were represented by the contributions of Sahroni et al. from Taiwan and Indonesia with Hybrid Nanomaterials for reversible lithium storage based on MoS2, Szabó et al. from Hungary and Switzerland present a paper on Titanium based nanomaterials for catalysis applications, Martinez et al. studied several Pt-Co Nanoparticles for CO oxidation, and finally the use of TiO2/Au nanoparticles for hydrogen generation via photocatalysis was explored by May-Masnou et al. from Spain.

In summary, this Research Topic have explored the state-of-the-art and beyond in the rational design of nanoparticles and nanocomposites, based on metallic, polymeric, or soft raw materials, as well as their different applications in medicine, imaging, drug delivery, catalysis, energy storage, or sensing.

For all future technological applications, the rational design of these multifunctional nanomaterials is critical, and in many cases will be controlled by the organic and inorganic chemical reactions involved during the production. The success of their applications relies directly on the photophysical and chemical properties created in the final material, including the emission of light or colourimetric responses, water solubility, selectivity, sensitivity, stability, thermal stability, functionalization, etc.

## Author Contributions

All authors listed have made a substantial, direct and intellectual contribution to the work, and approved it for publication.

### Conflict of Interest Statement

The authors declare that the research was conducted in the absence of any commercial or financial relationships that could be construed as a potential conflict of interest.

## Footnotes

^1^The International Caparica Symposium on Nanoparticles and Nanomaterials and Applications (ISN2A), is celebrated biannually in Caparica-(Portugal), is a high-quality forum to share innovative solutions to the many challenges created by human activity using and producing nanomaterials.

